# Population scale nucleic acid delivery to *Caenorhabditis elegans* via electroporation

**DOI:** 10.1093/g3journal/jkab123

**Published:** 2021-04-19

**Authors:** Anastasia S Khodakova, Daniela Vidal Vilchis, Dana Blackburn, Ferdinand Amanor, Buck S Samuel

**Affiliations:** 1 Alkek Center for Metagenomics and Microbiome Research and Department of Molecular Virology and Microbiology, Baylor College of Medicine, Houston, TX 77030, USA; 2 SMART Program, Baylor College of Medicine, Houston, TX 77030, USA

**Keywords:** *C. elegans*, nucleic acid delivery, electroporation, genetics

## Abstract

The free-living nematode *Caenorhabditis elegans* remains one of the most robust and flexible genetic systems for interrogating the complexities of animal biology. Targeted genetic manipulations, such as RNA interference (RNAi), CRISPR/Cas9- or array-based transgenesis, all depend on initial delivery of nucleic acids. Delivery of dsRNA by feeding can be effective, but the expression in *Escherichia coli* is not conducive to experiments intended to remain sterile or with defined microbial communities. Soaking-based delivery requires prolonged exposure of animals to high-material concentrations without a food source and is of limited throughput. Last, microinjection of individual animals can precisely deliver materials to animals’ germlines, but is limited by the need to target and inject each animal one-by-one. Thus, we sought to address some of these challenges in nucleic acid delivery by developing a population-scale delivery method. We demonstrate efficient electroporation-mediated delivery of dsRNA throughout the worm and effective RNAi-based silencing, including in the germline. Finally, we show that guide RNA delivered by electroporation can be utilized by transgenic Cas9 expressing worms for population-scale genetic targeting. Together, these methods expand the scale and scope of genetic methodologies that can be applied to the *C. elegans* system.

## Teaser

Genetic manipulations, like RNAi, CRISPR/Cas9- and array-based transgenesis depend on initial delivery of nucleic acids. Microinjection is effective for germline delivery, but limited by targeting each animal individually. We show that population-scale electroporation of dsRNA results in RNAi silencing throughout the worm, including in the germline. Electroporation of guide RNAs in Cas9 expressing worms can also induce gene disruptions. Together, these methods can be useful and expand the scale and scope of genetic methodologies developed for the *C. elegans* system.

## Introduction

Understanding gene function is an essential task of modern biology. The nematode *Caenorhabditis elegans* is one of the most widely used and versatile animal models for studying nearly all aspects of animal biology (Corsi *et al.* 2015; Meneely *et al.* 2019). For many years *C. elegans* has proven to be an effective and powerful genetically tractable system for functional characterization of genes in a whole organismal context (Housden *et al.* 2017). First, *C. elegans* allows for a rapid analysis of gene function carried out via targeted RNA interference (RNAi)-based knock-down of gene expression (Timmons and Fire 1998; Conte *et al.* 2015). Second, transgenic animals bearing exogenous genes can be created via microinjection of DNA constructs into the animal’s gonad resulting in the formation of heritable extrachromosomal arrays (Berkowitz *et al.* 2008). Third, CRISPR/Cas9 genome editing tools have been developed for precise genomic manipulations that allow desired *C. elegans* mutants to be engineered (Dickinson and Goldstein 2016). One of the critical steps for every genome manipulation pipeline is the delivery of nucleic acids inside the cell or animal. For *C. elegans*, microinjection of individual worms is a crucial step in the delivery of exogenous material. Microinjection remains the most time- and labor-intensive procedure for most *C. elegans* laboratories, whereas many other methods and approaches have been developed for different cellular and organismal systems (Alsaggar and Liu 2015). Among others, electroporation has been recognized as a powerful and quick method for simultaneous nucleic acid transfer in large populations of bacterial, yeast and mammalian cells (Young and Dean 2015). The electric pulse applied to the cell destabilizes its membrane and causes the formation of transient pores allowing exogenous material such as DNA, RNA, and proteins to enter the cell. Electroporation can also be used for introduction of exogenous material into entire tissues of the whole organism—*e.g.*, electroporation of DNA in zebrafish (Kera *et al.* 2010), Xenopus (Haas *et al.* 2002), or silkworms (Ando and Fujiwara 2013). However, this delivery method has not yet been applied to *C. elegans* animals. In this study, we demonstrate the feasibility and potential of the electroporation-based delivery of nucleic acids in *C. elegans* at a population scale. We show that electroporation-based delivery of double-stranded RNA (dsRNA) triggers RNAi gene silencing pathways inside *C. elegans*. This protocol is accomplished at the scale of hundreds of animals, making it broadly applicable and useful for nucleic acids delivery. Finally, we show in proof-of-principle studies that electroporation-mediated delivery of single-stranded guide RNA (gRNA) molecules can be utilized to disrupt genes in the progeny of Cas9 expressing animals. Together, we anticipate electroporation-based methods to greatly enhance the scope and scale of genetic targeting in this already robust genetic system.

## Materials and methods

### Worm strains and maintenance

All strains were cultured at 20° on Nematode Growth Medium (NGM) plates seeded with *Escherichia coli* strain OP50. Mutant strains VC1119 *[dyf-2&ZK520.2(gk505) III]* (referred as *[sid-2(gk505) III]* in current study) and HC196 *[sid-1(qt9) V]* were obtained from the Caenorhabditis Genetic Center. Transgenic GR1403 *[Is(sur-5::gfp) I; eri-1(mg366) IV]* strain was a kind gift from Gary Ruvkun. The BIG0105 *[Is(sur-5::gfp) I]* strain was produced by crossing GR1403 with the Samuel lab stock of N2. Strains BIG0106 *[sid-1(qt9) V; Is(sur-5::gfp) I]* and BIG0107 *[sid-2(gk505) III; Is(sur-5::gfp) I]* were generated by crossing of HC196 and VC1119 mutants with BIG0105 strain. Transgenic strain EG9888 that stably expresses Cas9 in the germlines of animals was kindly gifted by Dr. Matthew Schwartz and Dr. Erik Jorgensen [*W01A8.6(oxTi—[Pmex-5::cas9(+ smu-2 introns), Phsp-16.41::Cre, Pmyo-2::2xNLS-CyOFP + lox2272])I*]. A complete list of worm strains used and prepared in this study can be found in Supplementary Table S1.

### Synchronization

Nematodes were synchronized by bleaching and allowed to hatch overnight in M9 buffer (Stiernagle 2006). Density of the L1 larvae population was then measured by microscopy.

### Production of dsRNA

PCR products corresponding to *gfp*, *dpy-13*, *nhr-23*, and *pos-1* genes were generated with T7 primer (5′-AATACGACTCACTATAG-3′) and vectors isolated from the RNAi *E. coli* clones, using the following cycling conditions: 98° 15 s, 55° 15 s, 72° 60 s for 30 cycles. PCR product purification was performed according to the manufacturer’s protocol with QIAquick PCR Purification Kit (Qiagen, Cat. No. 28104). Purified PCR products were then used as templates for *in vitro* transcription per AmpliScribe T7 High Yield Transcription Kit (Epicentre Technologies, Cat. No. AS3107) specifications to obtain dsRNAs.

### Production of guide RNA

Production of the short gRNA (100 nt in length) specific to *dpy-10* gene was performed according to the protocol described in (Hwang *et al.* 2013). In brief, a plasmid encoding gRNA (targeting *dpy-10*) was constructed as follows: pDR274 vector for *in vitro* gRNA production (a gift from Keith Joung, Addgene plasmid # 42250; https://www.addgene.org/42250/; RRID: Addgene 42250) containing a T7 promoter upstream of gRNA scaffold sequence was digested with BsaI enzyme (NEB, Cat. No. R3733S). It was then used as a backbone for cloning the annealed oligonucleotides (dpy-10T: 5′-TAGGGCTACCATAGGCACCACGAG-3′; dpy-10B: 5′-AAACCTCGTGGTGCCTATGGTAGC-3′), containing *dpy-10* protospacer sequence (5′-GCTCGTGGTGCCTATGGTAG-3′). The sequence verified expression vector was then digested with HindIII enzyme (NEB, Cat. No. R3104S) and used as a template for *in vitro* transcription of gRNA by AmpliScribe T7 High Yield Transcription Kit (Epicentre Technologies, Cat. No. AS3107).

### Electroporation of L1 worms with dsRNA

An aliquot of the synchronized worms was spun down at 500 rcf for 2 min to provide approximately 250 worms (unless otherwise specified) in a volume of 5 μl after the centrifugation. Then 5 μl of worms were mixed with 40 μl of electroporation buffer (Gene Pulser Electroporation buffer, Biorad, Cat. No. 1652676) in 1.5 mL tubes, and allowed to incubate on ice for 5 min. An aliquot of 5 μl of purified dsRNA (10 μg/μl) was added to the worms just before the electroporation, mixed by pipetting, and transferred to 0.2 cm pre-chilled electroporation cuvettes (Biorad, Cat. No. 1652082). Animals were electroporated at 300 V for 10 ms (unless otherwise specified) by square-wave single pulse using a Bio-Rad Gene Pulser (BioRad, Cat. No. 1652660). Immediately after the electroporation, worms were washed with 1 mL of pre-chilled M9 buffer, transferred into 1.5 mL tubes and centrifuged for 2 min at 500 rcf. Supernatants were discarded and animals were then transferred to *E. coli* OP50 seeded plates and cultured at 20° for 48 h.

### Electroporation of L4 and young adult animals

Synchronized L1 larvae worms were cultured on OP50 plates at 20° until L4 (55 h) or Young Adult (70 h) stage. Then worms were washed off the plate with M9 buffer, followed by two additional washes in the same buffer to eliminate bacteria. Electroporation procedure for L4/YA worms was performed the same way as described for L1 worms. After the electroporation of dsRNA worms were allowed to recover for 48 h and then imaged. Progeny from the electroporated animals were collected for the first 24 h post electroporation, then adult worms were transferred to separate plates daily. Progeny development was monitored for 48 h (unless otherwise specified) and worms were imaged. After the electroporation with gRNA, worms were transferred on OP50 plates in groups of five worms per plate for recovery and progeny production. All F1 progeny with desired phenotype were then separated and maintained individually.

### Image acquisition and analysis

Microscopy-based analyses were used to count animals, measure body size and GFP fluorescence intensity. For imaging, worms were washed off the OP50 lawn with M9 buffer containing 20 mM of NaN_3_, washed with the same buffer two times to remove bacteria and then transferred to wells of a 96-well plate or glass slide with a 2% agarose pad. Animals were imaged using the Eclipse Ti-5 fluorescence microscope (Nikon) with 4× and 10× and 20× magnification under nonsaturating conditions using digital Z-stacking mode. Analysis of imaging data was performed using Fiji software (Schindelin *et al.* 2012) and custom-written MATLAB (Mathworks) scripts (Supplementary File S1). A minimum of 50 animals were analyzed per group for worm body length measurement and GFP fluorescence (unless otherwise specified). Worm GFP fluorescence was calculated by dividing the sum of GFP intensities of all pixels over the total pixel number for each worm. Then the background fluorescence, calculated as average fluorescence intensity of all pixels in a region without worm, was subtracted from worm fluorescence. GFP fluorescence per worm is defined in arbitrary units (a.u.). Time-lapse bright-field images of live worms with Dumpy and Roller phenotypes were used to create.mp4 video files of the worm’s movement (Supplementary File S2).

### Genotyping and Illumina sequencing

Genotyping of generated BIG0106 *[sid-1(qt9) V; Is(sur-5::gfp) I]* and BIG0107 *[sid-2(gk505) III; Is(sur-5::gfp) I]* transgenic strains was performed using single worms PCR and primers listed in Supplementary Table S2 followed by Sanger sequencing confirmation of generated PCR products. In order to identify presence of CRISPR editing in *dpy-10* gene after the gRNA electroporation, single worm PCR products were analyzed by Illumina sequencing using 2 × 250 bp pair-end run. Primers were designed to generate 450 bp PCR product with gRNA target sequence located in the middle of the amplicon. Worms were lysed in DNA Quick Lysate (Epicentre Technologies, Cat. No. QE09050) for 1 h at 60° and the lysate was then used as a template for PCR with Q5 Hot Start High-Fidelity DNA Polymerase (NEB, Cat. No. M0493S). PCR products were purified using QIAquick PCR Purification Kit (Qiagen, Cat. No. 28104). Barcoded library production and Illumina sequencing runs were provided by GENEWIZ. Two FASTQ files (R1 and R2) were generated for each sample (Supplementary File S3), and subsequently analyzed using Cas-Analyzer online tool (Park 2017).

### Statistical analysis

Comparison of multiple groups was performed using the analysis of variance (ANOVA) with Bonferroni correction. *P*-values < 0.05 were considered statistically significant. All experiments were performed at least two independent times.

### Data availability

All *C. elegans* strains, primers, and plasmids described in this study are available upon request. Supplementary materials are available at figshare: https://doi.org/10.25387/g3.13082270. Raw data (Supplementary File S4), scripts used for analyses (Supplementary File S1), and sequencing datasets (Supplementary File S3) can be found in Supplementary files.

## Results

### Initial considerations in development of an electroporation pipeline for *C. elegans*

**Figure 1:**
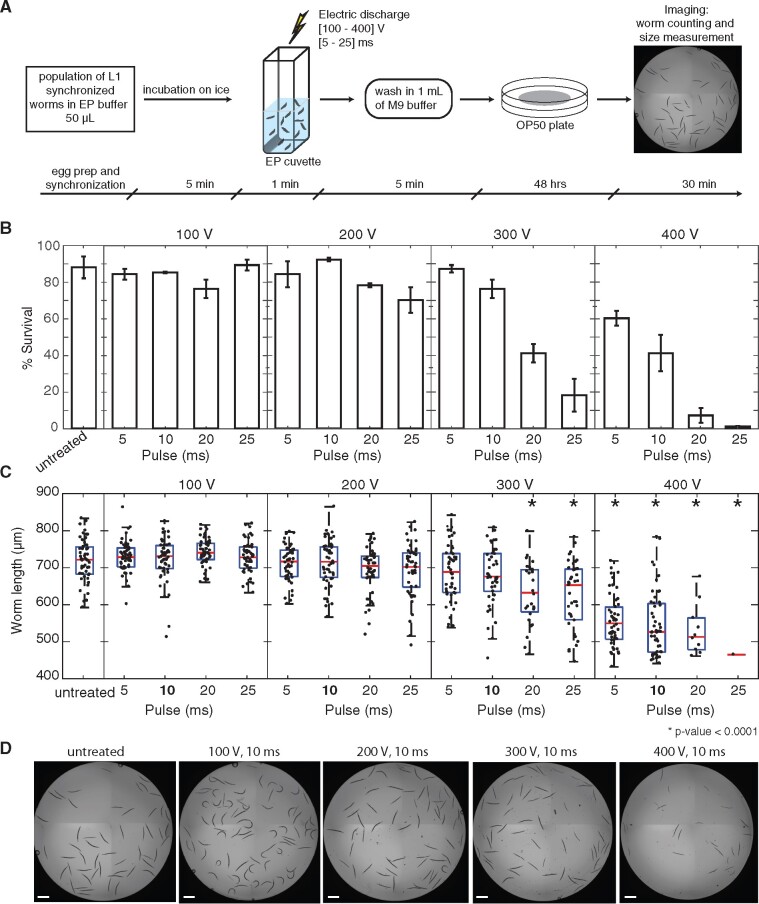
Optimization of electroporation conditions for *C. elegans* viability. General pipeline (A) of the electroporation procedure starts with the preparation of L1 synchronized worms (∼250), which are then mixed with electroporation buffer 80% in chilled cuvettes. After electroporation, worms are washed with 1 mL of M9 buffer and collected by centrifugation at 500 rcf for 2 min, then transferred to *E. coli* OP50 seeded plates to grow at 20° for 48 h. Animal survival rates (B) and body lengths (C) varied based on the electroporation conditions applied. The evaluation was performed using N2 animals for each pair of electroporation parameters with an electroporation pulse duration ranging from 5 to 25 ms and voltage ranging from 100 to 400 V. For (b), data were normalized to the initial calculated input number of worms (number of L1 worms was microscopically counted in 5 μl of the stock suspension before distribution between the experimental tubes). Animals placed in electroporation buffer without electric discharge were used as “untreated” controls. Worm survival rates: mean ± SD (standard deviation)% of two independent experiments. Body length measurements: here and on all other figures, if not stated otherwise, red lines indicate means, blue boxes show 25th and 75th percentiles, whiskers show the data distribution range. *P-values < 0.05 were considered statistically significant (ANOVA test with Bonferroni correction). Representative images (D) of worm populations exposed to electric discharges of different voltages (10 ms pulses) demonstrate the pronounced effect of the electroporation procedure on animal viability. Scale bar = 500 μm.

Based on applications in other systems, we first established a reliable and robust pipeline for electroporation of *C. elegans* (outlined in Figure 1A) that could serve as a basis for further optimization. Briefly, one part of the worm suspension with the desired number of worms is mixed with one part of the nucleic acid solution and eight parts of the electroporation buffer on ice to preserve the integrity of nucleic acids. The mixture then is transferred into the cuvette and electroporated under desired conditions. In this study, we electroporated worms in 50 μl of the final solution. In total, the electroporation procedure was rapid (15 min) and tolerated by the animals.

### Optimization of electroporation conditions for nucleic acids delivery while preserving animal viability

The efficiency of *in vivo* electroporation as a delivery tool is represented by an intersection of two key metrics: (1) maximum viability of worms under applied electroporation conditions and (2) the degree of material delivery itself. During electroporation, an electrical pulse is applied across the animal’s body with the assumption that some tissues may be more impacted than others. The cuticle, a multi-layered collagen outer tissue akin to our skin, provides considerable protection for the worm’s body and is likely to be a strong barrier for the electric pulses to bridge. To address these challenges, we sought to identify optimal electroporation parameters, in particular—pulse voltage and pulse length, that minimize adverse effects on worm physiology and maximize potential for nucleic acid delivery. These parameters were tested pairwise across a range of conditions for their impact on survival and developmental rates on populations of L1 synchronized N2 animals (∼250). Microscopy-mediated worms’ assessment was performed after 48 h of recovery on *E. coli* lawns. Robust animal viability (number of alive worms was counted 48 h after electroporation procedure and normalized to the initial calculated input number of worms taken) was observed (>70%) at lower voltages (100–200 V) regardless of the pulse duration, and up to 10 ms pulses for 300 V treatments (Figure 1B). Beyond these conditions, treatment of worms at or above 300 V for longer than 20 ms significantly decreased animal survival rates (Figure 1B). It is worth noting that electroporation procedure comprising multiple worm transfers and washing steps implies some degree of worm loss, which in current experiments was estimated to be up to 18% (Figure 1B, untreated). Based on measurements of animal length and vulval morphology, similar combinations of high voltage and long duration of the electric pulse caused significant developmental delays in electroporated worms compared to the untreated control animals (Figure 1, C and D). Fecundity rates of electroporated L1 worm populations under favorable conditions (at or below 300 V and 10 ms) also appeared to be similar to untreated controls (data not shown). Thus, treatment of worms with 300 V for pulse durations up to 10 ms minimizes adverse effects on animal viability and developmental timing while maximizes potential for material delivery. As we were also interested in the delivery of nucleic acids to the germlines, we performed similar optimization of electroporation parameters on L4 animals (N2) that are closer to reproductive maturity. Using the same matrix of voltage and pulse duration times as for L1 animals before, favorable viabilities remained >70% up to 400 V 10 ms (Supplementary Table S3). Despite the apparent resilience in L4 animals from a viability perspective, we observed a collapse of one or more of the gonads in up to ∼15% of the cases starting from 300 V and 20 ms and onward, and as a consequence, a decrease in fecundity rate (data not shown).

### Evaluation of the effectiveness of electroporation of dsRNA in *C. elegans* populations

**Figure 2:**
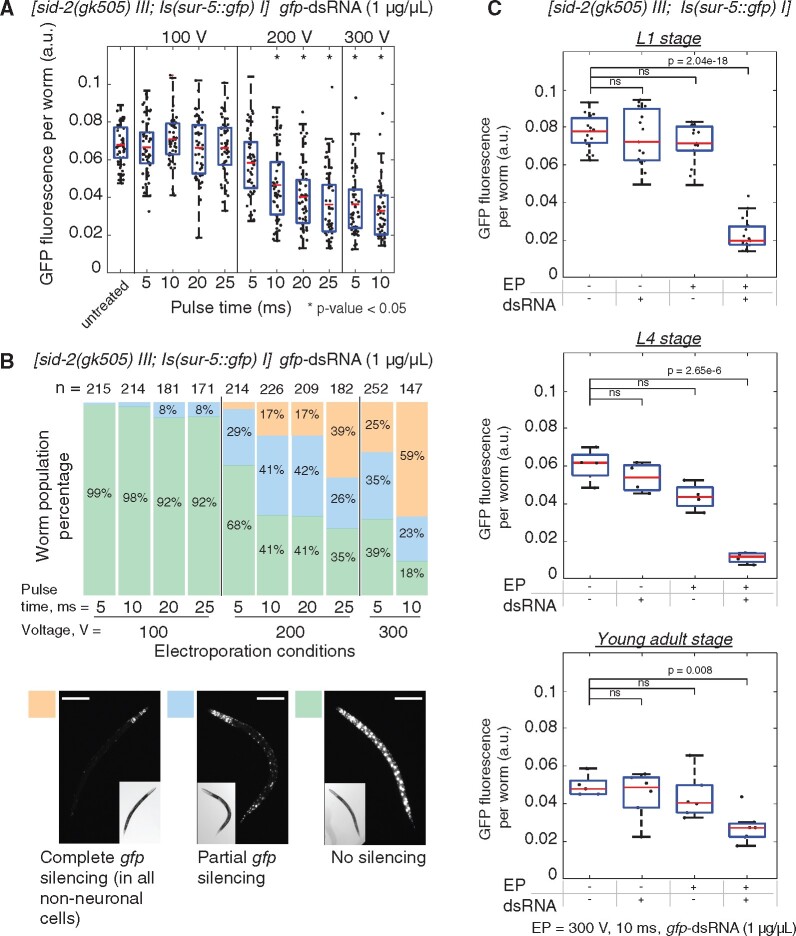
Identification of electroporation conditions for efficient delivery of dsRNA in *C. elegans*. To evaluate the effectiveness of nucleic acid delivery into animals, we used highly sensitive RNAi-mediated silencing of a GFP transgene following electroporation of dsRNA. (A) Synchronized L1 populations of BIG0107 *[sid-2(gk505) III; Is(sur-5: gfp) I]* worms (∼250) were electroporated with *gfp*-dsRNA of 1 μg/μl using favorable electroporation conditions. Animals placed in electroporation buffer without dsRNA or electric discharge were used as “untreated” controls. For each condition, GFP fluorescence intensity of worms (*n* = 50) was measured in arbitrary units (a.u.). Asterisk (*) indicates groups where significant *gfp* silencing compared with the untreated control was observed (*P*-value < 0.05, ANOVA test with Bonferroni correction). Results of body length comparison between worms electroporated at different conditions and untreated worms showed no significant differences (see Supplementary Figure S1A). (B) Three phenotypic categories of animals were scored in each condition group, including worms with “No silencing”, “Partial *gfp* silencing,” and “Complete *gfp* silencing” (all but neuronal cells). *n* = number of worms scored. Representative images of worms from each category are shown, scale bar = 100 μm. The electroporation parameters of 300 V 10 ms with the highest percentage in “Complete *gfp* silencing” category (59%) were chosen as the most efficient. (C) Populations of BIG0107 animals at L1, L4, and YA stages were electroporated with *gfp*-dsRNA of 1 μg/μl using electroporation conditions of 300 V 10 ms. Levels of GFP fluorescence were measured in 48 h after electroporation and compared to the control worms including untreated animals [electroporation (−)/dsRNA (−)], electroporated animals without dsRNA [electroporation (+)/dsRNA (−)], and animals incubated with dsRNA [electroporation (−)/dsRNA (+)] (*P*-values are noted, ANOVA test with Bonferroni correction). No significant differences in worm body lengths were observed (see Supplementary Figure S1, B–D).

Silencing by RNAi in *C. elegans* is a sensitive method for specific knockdown of gene expression (Conte *et al.* 2015), and when applied to fluorescent transgenes, RNAi provides a robust visual phenotypic readout of the degree of knockdown at a cellular level. In *C. elegans*, RNAi-mediated silencing can be achieved by feeding worms with *E. coli* expressing a gene-specific dsRNA (Timmons and Fire 1998) or via soaking of worms in a highly concentrated solution of dsRNA ranging from 0.5 to 5 μg/μl (Ahringer 2006). Ingested dsRNAs are recognized by the lumenal receptor SID-2 and subsequently engulfed (McEwan *et al.* 2012). Engulfed dsRNAs are released and spread into the cell cytosol (and throughout the animal) via SID-1 membrane channels (Wang and Hunter 2017). The presence of dsRNA in the cytosol triggers canonical RNA dependent RNA polymerase (RDRP)-based amplification and ultimately RNAi silencing of target genes (Shih and Hunter 2011). In order to test the effectiveness of electroporation, we utilized this highly sensitive system to identify animals and tissues that were effectively delivered dsRNAs. To do this, we used transgenic animals BIG0107 that both produce GFP ubiquitously in the nuclei of all somatic cells and lack the ability to take up dsRNA from the intestine. Synchronized L1 populations of animals were electroporated using favorable conditions identified above and monitored for *gfp* silencing as a proxy for effectiveness of dsRNA delivery. Though all treatments with 100 V did not result in silencing, we observed significant reductions in GFP fluorescence in animals treated with 200 V or greater compared to the untreated control (Figure 2A). Based on phenotypic analyses of the electroporated animals, we identified that treatments of animals with 300 V for 10 ms yielded the highest percentage of animals in the completely silenced (all but neuronal cells) category at nearly 60% (Figure 2B). Selected electroporation conditions were applied for *gfp*-dsRNA delivery to BIG0107 worms at different developmental stages, including L1, L4, and young adults (YA) (Figure 2C). We observed significant decreases in GFP fluorescence in electroporated animals at all stages compared to the controls and minor but nonsignificant impacts of electroporation on animal length in L4s and YAs. Together, these results identified effective conditions that allow the delivery of dsRNAs into *C. elegans* animals.

### Determination of the tissue distribution of dsRNA delivery in *C. elegans*

**Figure 3:**
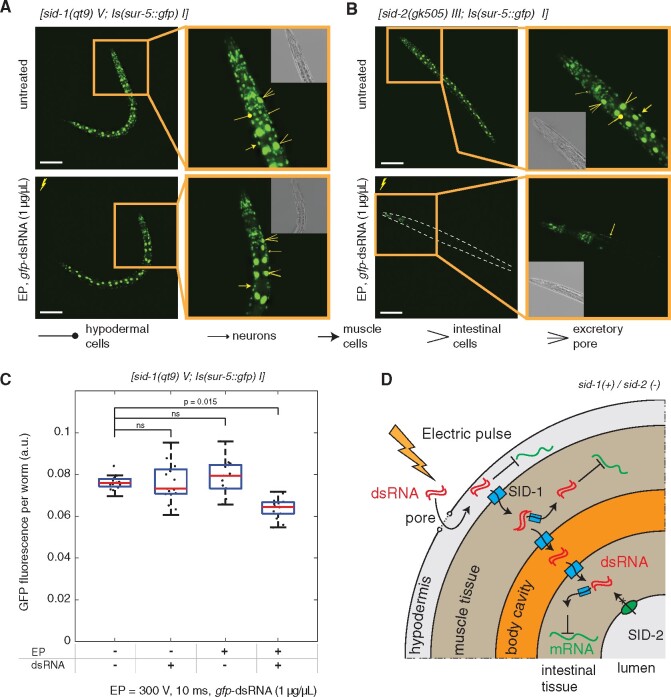
Evaluation of tissue distribution of RNAi silencing in electroporated animals. (A, B) Representative images of BIG0106 *[sid-1(qt9) V; Is(sur-5::gfp) I]* and BIG0107 *[sid-2(gk505) III; Is(sur-5::gfp) I]* worms were taken 48 h after the electroporation of L1 worm populations with *gfp*-dsRNA of 1 μg/μl using 300 V 10 ms conditions. Images of “untreated” control animals [electroporation (−)/dsRNA (−)] are presented for comparison. Scale bar = 100 μm. Additional images of these worms presented in three digital focal planes can be found in supplementary information (Supplementary Figures S3 and S4). (C) Levels of GFP fluorescence in *[sid-1(qt9) V; Is(sur-5::gfp) I]* worm strains (*n* = 15) after electroporation were compared to the controls worms (*P*-values are noted, ANOVA test with Bonferroni correction). No significant differences in body lengths between electroporated and control worms were observed (Supplementary Figure S2). (D) Schematic of the presumed routes of dsRNA transport in *sid-1*(+)/*sid-2*(−) animals highlights hypodermal entry as a primary site of initial dsRNA delivery by electroporation, followed by spread to other tissues in a SID-1-dependent manner.

With conditions for delivery optimized, we next sought to identify the breadth of tissues that could be effectively electroporated. To test this, we utilized a similar reporter system together with the BIG0106 mutant defective in systemic RNAi, as SID-1 membrane channels facilitate spread of dsRNAs between tissues and into cells (Whangbo *et al.* 2017). In this manner, *gfp* silencing should only be observed in those tissues and cells where *gfp*-dsRNA was directly delivered into the cell cytoplasm. Loss of systemic RNAi in these mutants predictably reduced the overall level of *gfp* silencing (Figure 3). Microscopic assessment of the animals indicated that silencing within hypodermal cells likely accounted for the majority of the significant decreases of GFP expression observed in *sid-1* mutants compared to controls (Figure 3, A and C). These results do not exclude the possibility of delivery to other tissues, but suggest that the degree of delivery may be less efficient and would require additional optimization for silencing to occur. The presence of a large proportion of worms with partial *gfp* silencing (Figure 2) also suggests that the impact of the electric pulse along the animal body may not be uniform and depends on worm position in the cuvette that could lead to observed *gfp* silencing variations both between and within animals. Together these results indicate that electroporation delivers *gfp*-dsRNA most efficiently to hypodermal cells and then spreads to other tissues in a SID-1-dependent manner (Figure 3, B and D).

### Dose dependent delivery of dsRNA by electroporation

**Figure 4:**
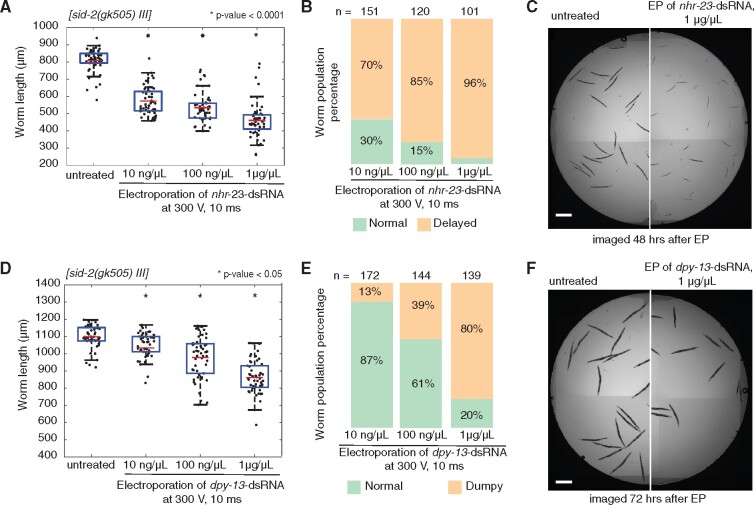
Efficiency of electroporation-driven gene silencing of endogenous genes is dose dependent. In order to test the effectiveness in nontransgenic animals, we targeted endogenous hypodermally expressed genes with robust RNAi phenotypes, *nhr-23* [larval arrest, (A–C)] and *dpy-13* [shortened body size, (D–F)]. (A) Impact of electroporation of *nhr-23*-dsRNA on the development of *sid-2(gk505)* worms treated at L1 stage and imaged after 48 h. Red lines indicate means, blue boxes show 25th and 75th percentiles, whiskers show the data distribution range. (B) Proportion of animals scored as having either “normal” or “delayed” development after electroporation. (C) Representative image of worms show the *nhr-23* silencing effect at 1 μg/μl of dsRNA (right image), when compared to untreated worms (left image). Scale bar = 500 μm. (D) Impact of electroporation of *dpy-13*-dsRNA on body size of *sid-2(gk505)* worms treated at L1 stage and imaged after 72 h. Red lines indicate means, blue boxes show 25th and 75th percentiles, whiskers show the data distribution range. (E) Proportion of animals scored as “normal” or “dumpy” after electroporation. (F) Representative images of worms demonstrate the *dpy-13* silencing at 1 μg/μl of dsRNA (right image) in comparison with untreated control worms (left image). Scale bar = 500 μm. Asterisk (*) indicates groups with significant gene silencing compared to the untreated control (ANOVA test with Bonferroni correction).

RNAi mediated silencing in *C. elegans* occurs in a dose-dependent manner (Whangbo *et al.* 2017), which can be particularly useful when testing functions of essential genes. Because the experiments outlined above utilized highly concentrated levels of dsRNA (1 μg/μl), we next sought to identify whether we could control degree of silencing by titrating the levels of dsRNA targeting native genes delivered to the VC1119 animals. To test this, we selected two native genes expressed in the hypodermis with readily quantifiable size-based phenotypes, *nhr-23* (developmental arrest, Kouns *et al.* 2011) and *dpy-13* [dumpy (von Mende *et al.* 1988)], to trigger silencing by different levels of dsRNA concentration (10, 100 ng/μl, and 1 μg/μl). For each gene, we observed dose-dependent, electroporation-driven ranges in silencing depending on the amount of dsRNA in solution (Figure 4, A and D). Notably though, 100-fold less concentrated *nhr-23*-dsRNA was able to cause developmental arrests in 70% of animals compared to 96% for animals treated with 1 μg/μl of *nhr-23*-dsRNA (Figure 4, B and C). While for *dpy-13*, we observed a lower penetrance of the dumpy phenotype and more gradual decrease in the average worm size with the increase of *dpy-13*-dsRNA concentration (Figure 4, D–F). Additional analyses indicate no influence of the applied electroporation procedure on animal body length in comparison with the untreated control worms (Supplementary Figure S5). Together, these results illustrate that electroporation of dsRNA can titrate levels of gene silencing with minimal levels of starting dsRNA material.

### Germline delivery and transmission of electroporated dsRNA to progeny

**Figure 5:**
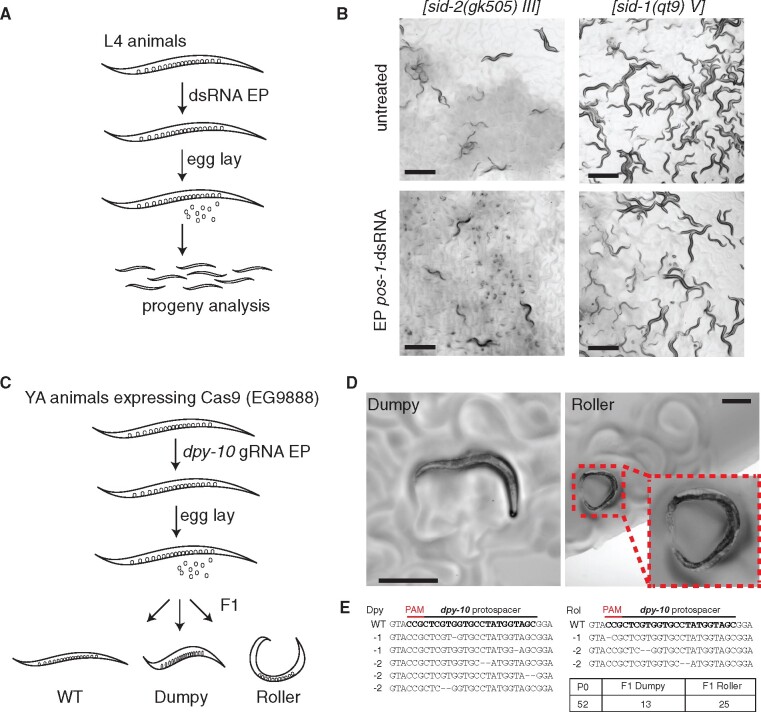
Evaluation of electroporation for delivery to the animal germline and progeny. To further test the utility of this approach, we sought to identify whether we could, first, stimulate RNAi knockdown of endogenous gene *pos-1* expressed in germline with robust phenotype (embryonic lethality) and, second, deliver guide RNA (gRNA) for CRISPR/Cas9-mediated genome editing of the endogenous *dpy-10* gene. (A) Schematic of *pos-1*-dsRNA delivery to L4 worms by electroporation (300 V for 10 ms, 1 μg/μl of dsRNA in electroporation buffer with final volume of 50 μl) followed by phenotypic analysis of progeny. Animals were allowed to lay eggs for 24 h, removed from the lawn, and the proportion of hatched progeny was determined after 48 h. (B) Representative images of effective electroporation-mediated delivery of *pos-1*-dsRNA to *sid-2(gk505)* animals. Scale bar = 500 μm. Proportions of hatched/unhatched embryos from individual worms after *pos-1*-dsRNA electroporation compared to control animals demonstrated in Supplementary Figure S6. (C) Schematic of delivery of *dpy-10* gRNA to Young Adult (YA) animals by electroporation (300 V for 10 ms, 1 μg/μl of RNA in electroporation buffer with final volume of 50 μl) followed by phenotypic screening of progeny for evidence of genome editing (Dumpy or Roller). (D) Representative images of successful electroporation of *dpy-10* gRNA in EG9888 animals that resulted in F1 progeny with visible Rol and Dpy phenotypes. Scale bar = 300 μm. (E) Illumina sequencing-based confirmation of Cas9-mediated mutations.

Next, we examined whether electroporation could be used to deliver material to the germline of animals. We expected the most efficient transmission of dsRNAs to occur in animals that are at or near reproductive maturity (*i.e.*, L4 stage or older, Marré *et al.* 2016). To test whether dsRNA can target the germline, populations of L4 animals VC1119 were electroporated with a germline-specific *pos-1*-dsRNA of 1 μg/μl (Figure 5A), as efficient silencing of the *pos-1* gene produces a robust embryonic lethal phenotype (?). After 24 h adult animals were removed from the plate and the progeny were scored for hatching after an additional 48 h. We observed reproducible delivery and efficient *pos-1* silencing in the majority (50% or greater) of animals as evidenced by the prevalence of unhatched eggs from electroporated animals compared to those of untreated control animals both as a population (Figure 5B) and as individuals (Supplementary Figure S6). Despite the greater level of variation in L4 animals, these results indicate that once delivered hypodermally the dsRNA can spread and silence effectively in the germline, which we are not able to observe with *sur-5::gfp* strains due to intrinsic germline silencing of *gfp* transgenes. In addition, we also tested *sid-1(qt9)* mutants defective in systemic RNAi and observed no difference in progeny development derived from the electroporated population of worms compared to the control worms (Figure 5B). Together, these studies indicate transmission of electroplated dsRNA to the germlines.

### Evaluation of electroporation to deliver guide RNA to germlines for Cas9-mediated genome editing

Since we demonstrated that we could deliver dsRNAs to the germline, we tested whether delivery could be extended to guide RNAs (gRNAs) for CRISPR/Cas9 based genome editing. Typically, gRNAs are injected along with additional components into the germlines of animals one-by-one to target disruption of specific genes (Prior *et al.* 2017). In this study, we took advantage of transgenic worms stably expressing *cas9* in the germline [EG9888 *[W01A8.6(oxTi—-[Pmex-5::cas9(+ smu-2 introns), Phsp-16.41::Cre, Pmyo-2::2xNLS-CyOFP + lox2272])I*]; unpublished, a gift from Dr. Matthew Schwartz and Dr. Erik Jorgensen) that should only need introduction of gRNAs to facilitate targeting. We then chose to deliver a well-characterized and robust gRNA targeting *dpy-10* that is commonly used as a co-CRISPR marker for CRISPR/Cas9 editing during microinjection (Arribere *et al.* 2014). In order to ensure robust Cas9 production, we electroporated *dpy-10*-gRNA (1 μg/μl; 300 V and 10 ms) into a population of YA worms (*n* = 52) that were further divided and maintained on *E. coli* OP50 in groups of five worms per plate (Figure 5C). As additional controls, we included both soaking of EG9888 YA worms in *dpy-10*-gRNA solution (1 μg/μl) for 15 min to be aligned with conditions of electroporation procedure and continuous feeding the worms for two generations on HT115 *E. coli* producing *dpy-10*-gRNA; neither of these controls produced phenotypically altered progeny (data not shown). Electroporated *dpy-10*-gRNA was able to be successfully delivered in at least some of P0 worms in the population, which albeit at low levels resulted in F1 progeny production with Rol (*n* = 25) and Dpy (*n* = 13) phenotypes (Figure 5D; Supplementary File S2). F1 worms were singled and transferred to new OP50 plates in order to evaluate phenotype transmission in F2 worms. The observed phenotypic changes, however, were not heritable or lethal and more likely were only somatic in F1 animals, as F2 progeny did not retain their phenotypes. Consistent with this notion, single worm PCR followed by Illumina sequencing of F1 Rol and Dpy animals identified low indels frequency rates ranging from 0.2 to 1.3% with single and dinucleotide deletions (Figure 5E). NGS sequencing data was analyzed using Cas-Analyzer bioinformatic tool (Park 2017). Worth noting, in order to confirm functionality of *in vitro* produced gRNA, EG9888 animals were injected with *dpy-10*-gRNA followed by F1 progeny selection with Rol and Dpy phenotypes. F2 progeny from these animals inherited Rol and Dpy phenotypes and the presence of inherited edits in *dpy-10* locus in these worms was confirmed by single worm PCR followed by Sanger sequencing (data not shown). Together, these results suggest that electroporation-based delivery of gRNAs is possible, but further optimization is needed to increase the efficiency of genomic targeting moving forward.

## Discussion

We demonstrate that nucleic acids can be delivered via electroporation into *C. elegans* worms at several stages of life. Electroporation conditions were optimized to maximize animal viability and potential for material delivery. Using RNAi as a sensitive readout for delivery of dsRNA, we show that electroporation-mediated delivery of *in vitro* synthesized gene-specific dsRNAs resulted in RNAi silencing of both GFP-reporter transgenes and native genes, such as *nhr-23*, *dpy-13*, *pos-1*. Dose-dependent increase in electroporation-driven RNAi silencing was demonstrated with dsRNA concentrations ranging from 10 ng/μl to 1 μg/μl. The use of *sid-1(qt9)* and *sid-2(gk505)* mutants with *sur-5::gfp* transgene reporter allowed us to dissect the way electroporated dsRNA enters inside the worm body. Namely, electroporated dsRNA is delivered into the cytoplasm of hypodermal cells and distributed systemically by SID-1 RNA channel throughout the body and into germlines (Figure 3D). The proposed electroporation method of population scale dsRNA delivery is quick, easy and can be accomplished in 15 min compared to traditional 24–48 h needed for efficient RNAi by feeding and soaking (Ahringer 2006). Being able to pair host genetic knockdowns that do not require alterations in the physiology of the animal are key to the usefulness of the system regardless of the question being interrogated. Studies of *C. elegans* commonly rely on standard *E. coli* OP50 diet in the laboratory and on RNAi screenings where the other *E. coli* strain HT115 is used both as a diet source and a producer of dsRNA. It was found that these two *E. coli* strains differentially affect gene expression profiles in worms (Coolon *et al.* 2009) and influence on animal metabolism, physiology, development, behavior, immunity, and lifespan. For this reason, recent advances have led to the development of an *E. coli* OP50 RNAi strain (Neve *et al.* 2020). However, expanded appreciation for and widespread utilization of microbes from *C. elegans* natural microbiome (Zhang *et al.* 2017), each with their own impact on aspects of host physiology (Samuel *et al.* 2016), complicates this paradigm. Each strain would need its own RNAi library in order to properly examine the genetics of host-microbe interactions in these cases. Thus, we believe that electroporation as a bacteria-free dsRNA delivery method can mitigate the need to introduce another microbe into the mix (*E. coli*) in RNAi-based tests of host-microbe interactions. In addition, compared to RNAi silencing implemented via soaking, which is also a bacteria-free method, electroporation eliminates prolonged worm starvation or larval developmental arrest, which also has a pronounced effect on worm gene expression profiles particularly if completed early in life (Rechavi *et al.* 2014).

Beyond knockdowns, many effective strategies have been developed for precise genome editing of *C. elegans* (Dickinson and Goldstein 2016; Schwartz and Jorgensen 2016; Wang *et al.* 2018; Au *et al.* 2019; Whangbo *et al.* 2020). Nearly all of these strategies rely on low-throughput microinjection methods for delivery of nucleic acids mixtures. Here, we present proof-of-principle studies that electroporation may be a useful strategy for circumventing the microinjection step in these pipelines through population-scale delivery of guide RNAs in Cas9 expressing transgenic worms. We observed the Rol and Dpy phenotypes after electroporation of YA worms with *dpy-10* gRNA only in the F1 generation, suggesting that phenotypes were presumably caused by editing in somatic cells. Further studies will be needed to determine whether this is due to the delivery route that the electroporated gRNA reached the germline or other reasons. It has been shown that SID-1, a nonselective dsRNA transporter (Shih *et al.* 2009), also transports hairpin RNA molecules containing greater than 300 nucleotides single-stranded regions (Shih and Hunter 2011). Therefore gRNA, which contains hairpin structures in the scaffold or tracrRNA sequence, likely reaches the germline via the SID-1 dependent spreading from hypodermal cells after electroporation. In addition, previous studies have demonstrated that nonspecific import of dsRNA from the body cavity to proximal oocytes and embryos in mature worms also occurs along with the RME-2 mediated uptake of small yolk granules (Marré *et al.* 2016), suggesting potential involvement of the mechanism into the electroporated gRNA transportation. The somatic editing in F1 generation of worms is believed to be a consequence of residual Cas9 activity in the fertilized embryos, which is commonly observed after microinjections of CRISPR/Cas9 complex in worm’s syncytial gonads (Cho *et al.* 2013). This explanation fits well to our experimental results given that in EG9888 transgenic strain *cas9* is expressed under cytoplasmic germline *mex-5* promoter which remains active in fertilized eggs as well (Tenlen *et al.* 2008). Deeper understanding of the electroporation-mediated delivery process and further adaptation of the method to facilitate transfer of nucleic acids to the germline aiming to generate heritable genome modification is needed. For instance, the encapsulation of the nucleic acids in liposomes prior to electroporation might be one of the options that could potentially enhance RNAi and CRISPR editing (Adams *et al.* 2019). Further, this approach may hold great utility for delivery of dsRNA via electroporation in other *Caenorhabditis* species/strains or other parasitic nematodes that are susceptible to RNAi by injection but not by feeding and soaking (Nuez and Felix 2012). It may be possible to engage this pathway for more efficient and timely transfer of gRNAs to the germline. Overall, we believe that our findings hold a promise for further development of population scale, electroporation-mediated delivery of nucleic acids into *C. elegans* for a wide variety of applications.
